# Neuroimaging and artificial intelligence for assessment of chronic painful temporomandibular disorders—a comprehensive review

**DOI:** 10.1038/s41368-023-00254-z

**Published:** 2023-12-28

**Authors:** Mayank Shrivastava, Liang Ye

**Affiliations:** 1 Adams School of Dentistry, University of North Carolina, Chapel Hill, NC USA; 2 Department of Rehabilitation Medicine, University of Minnesota Medical School, Minneapolis, MN USA

**Keywords:** Oral diseases, Dental diseases

## Abstract

Chronic Painful Temporomandibular Disorders (TMD) are challenging to diagnose and manage due to their complexity and lack of understanding of brain mechanism. In the past few decades’ neural mechanisms of pain regulation and perception have been clarified by neuroimaging research. Advances in the neuroimaging have bridged the gap between brain activity and the subjective experience of pain. Neuroimaging has also made strides toward separating the neural mechanisms underlying the chronic painful TMD. Recently, Artificial Intelligence (AI) is transforming various sectors by automating tasks that previously required humans’ intelligence to complete. AI has started to contribute to the recognition, assessment, and understanding of painful TMD. The application of AI and neuroimaging in understanding the pathophysiology and diagnosis of chronic painful TMD are still in its early stages. The objective of the present review is to identify the contemporary neuroimaging approaches such as structural, functional, and molecular techniques that have been used to investigate the brain of chronic painful TMD individuals. Furthermore, this review guides practitioners on relevant aspects of AI and how AI and neuroimaging methods can revolutionize our understanding on the mechanisms of painful TMD and aid in both diagnosis and management to enhance patient outcomes.

## Introduction

Pain is a multidimensional experience which involves sensory-discriminative, affective- motivational, and cognitive-evaluative components.^1,2^ Chronic pain exerts an enormous personal and economic burden with prevalence ranges between 11% and 40%.^3,4^ The rising rates of depression, anxiety, changes in work demands, life styles and behavior, obesity, sleep problems, genetics, and increased symptom awareness could all be the factors driving this increase.^2^ Chronic pain directly impacts individuals physiological and psychological states and can occur as a result of neuroplastic remodeling on various levels of nervous system, ranging from synaptic plasticity to reorganization of large scale neural networks which can lead to maintenance of pain even in the absence of nociceptive inputs.^5^

Individuals with chronic pain are typically diagnosed with one or more regional or widespread central pain conditions such as fibromyalgia, chronic pelvic pain, back pain, headaches, chronic fatigue syndrome, and temporomandibular disorders (TMD).^6^ TMD are cluster of musculoskeletal conditions which affect masticatory muscles and temporomandibular joint and/or associated structures.^7^ It afflicts ~10%–15% of the population at a clinically significant level, with symptoms severe enough to warrant a medical attention.^8^ Epidemiological studies have shown that TMD affects 5-12% of general population, with “Orofacial Pain Prospective Evaluation and Risk Assessment Study (OPPERA)” reporting an annual incidence of 3.9% in adults.^9,10^ Etiology of TMD are multifactorial involving both biomechanical and biopsychosocial factors. It is hypothesized dynamic interaction between peripheral and central nervous system contributes to the development of chronic painful TMD.^11–13^ Besides this other mechanism contributes to development and perpetuation of pain are abnormal autonomic functions, neuroendocrine system and triggers such as psychosocial stressors and emotional or physical trauma.^14,15^ TMD are difficult to diagnose and mange due to complexity of the disorder and limited understanding on underlying mechanisms. Furthermore, the factors that contributes to development and maintenance of chronic painful TMD are not fully elucidated. To investigate this, over the last decades researchers have been exploring how brain shapes in chronic painful TMD.^16–20^

Neuroimaging techniques, for instance functional and structural MRI methods have been widely used separately or combined to explore brain alterations in patients with chronic pain including TMD. To some extent neuroimaging investigations have disentangle the neural mechanism underlying chronic painful TMD.^21–23^ According to the previous investigations, somatosensory, limbic system and associative brain structures are considered to be involved in the pain system. These areas have been linked to number of characteristics of pain evaluation and experience including anticipation, affective processing of pain and anti- nociception. Accumulating evidence on painful TMD patients have revealed structural and functional changes in the pain related network including ascending trigeminal-thalamo-cortical pathway, lateral (sensory- discriminative areas) and medial systems (affective-cognitive and evaluative areas), antinociceptive pathway, salience network and default mode network (DMN) and motor system.^23^ However, the application of neuroimaging in identifying the patterns of altered brain function, structure and chemistry in individuals with chronic painful TMD is still in its development. Furthermore, there is a paucity of clinical research data on applications of neuroimaging methods in individuals with chronic painful TMD making it challenging in identifying diagnostic and prognostic brain markers in individuals with TMD.

**Fig. 1:**
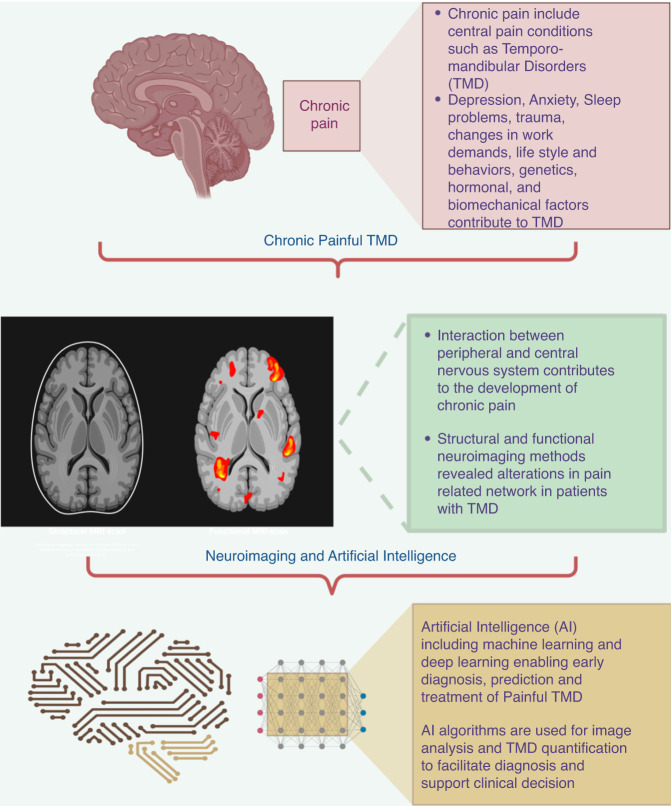
Overview of relationship between temporomandibular disorders, neuroimaging and AI

Over the last few decades informatics approaches, such as artificial intelligence (AI), have been called to help to address these challenges. AI is enabling early diagnosis, prediction, and treatment of chronic pain disorders that compromise brain health by advancing clinical translational imaging in particular neuroimaging.^24,25^ (Fig. 1). Machine learning (ML) and Deep learning (DL) which use artificial neural networks inspired by neuronal architectures are two class of AI, that have received extensive research.^24^ An increasing number of AI algorithms are being used in patient diagnosis, particularly for identifying and categorizing lesions like skin malignancy, brain tumors and dental diseases. For the diagnosis of painful TMD, a range of AI algorithms have recently been applied to imaging and non-imaging data.^25–27^ However, studies on the use of AI algorithms in patients with orofacial pain disorders specifically involving TMD are limited.^26,27^ Furthermore, the conclusion drawn from the previous studies on the use of AI and neuroimaging are limited by its specific research condition, including study design, approach of neuroimaging data analysis, disease subtypes, input data used for diagnosis, and outcomes measures for performance evaluation.

Therefore, main objective of this review is to familiarize investigators with the neuroimaging methods available for exploring chronic painful TMD as well as to highlight the neuroimaging studies that reveal structural and functional changes in the brain associated with painful TMD. It also discusses on how AI can be used to quantify TMD, identify brain patterns based on the neuroimaging data and enhance our understanding on unraveling the mechanism that contributes to pain chronification.

## Neuroimaging methods

Neuroimaging can be broadly characterized as structural imaging which aims to visualize the anatomy of central nervous system (CNS) and functional imaging which captures the brain neurophysiological or metabolic process. To date, TMD have been studied using different functional, structural and neurochemical imaging techniques such as fMRI, proton magnetic resonance spectroscopy (H-MRS) and arterial spin labeling (ASL) and positron emission tomography (PET). Additionally, electroencephalography (EEG) and magnetoencephalography (MEG) have shed light on how brain reacts to nociceptive stimuli^20^ (Table 1).

**Table 1:** Different neuroimaging methods used to study brain

Summary of neuroimaging methods discussed in the review			
Neuroimaging methods	Nature of activity measured	Strengths	Limitations
**fMRI BOLD**	Detect activity in the brain as determined by oxyhemoglobin and deoxyhemoglobin.	High Spatial resolution, Non-invasive, can measure cognitive process	Indirect measure of neural activity, use of artificial stimulus may not reflect pain.
**fMRI-ASL**	Technique use arterial water as an endogenous tracer to measure cerebral blood flow.	Task or pain stimulus not needed /representative of ongoing clinical pain.	Indirect measure of neural activity
**MRI-DTI**	Measures diffusion of water in the brain	Increase understanding of neural networks	Data artifacts, Limited resolution
**MRI-Structural**	Structural information delineate the body of white and gray matter using voxel-based parameters.	Provide excellent structural information, Measures assessment of diseases of overtime.	High costs
**H-MRS**	Measures proton bind-organic compound, Provide metabolic activity.	Easy metabolite identification	Low sensitivity and resolution
**PET**	Measures cerebral blood flow, glucose metabolism, oxygen uptake using radionuclides.	Measures cerebral metabolism, ligands and drug binding, Receptor mapping	Expensive, use radio-tracer, technical complexity.
**EEG**	Direct recoding of electrical changes of brain activity associated with cognitive task and behavior.	Comparatively lower cost, signals generate direct from neural activity, non-invasive.	Use of artificial stimulus many do not reflect clinical pain.
**MEG**	Event related potential detected by magnetic field.	Signals generated directly from neural activity, Good spatial resolution.	Expensive, Needs a magnetic shielded room.

*fMRI* functional magnetic resonance imaging, *BOLD* blood oxygen level dependent, *ASL* arterial spin labeling, *MRI–DTI* magnetic resonance imaging–diffusion tensor imaging, *H-MRS* proton- magnetic resonance spectroscopy, *PET* positron emission tomography, *EEG* electroencephalography, *MEG* magnetoencephalography

The most commonly used modality for studying painful TMD is fMRI that measure and analyze so called BOLD (Blood oxygen level dependence) effect. BOLD effect indicates changes in oxyhemoglobin deoxyhemoglobin ratio driven by increase in regional blood flow and volume that corresponds to the increase in brain activity. However, BOLD signal is merely an indirect neural activity, and it is still unclear how excitatory and inhibitory neural activity influences the bold signal.^5^ Additional approaches of BOLD fMRI are the task related conventional method used to reveal brain regions that are functionally involved in specific tasks such as painful stimuli or stimulus evoked pain such as allodynia. Other approach known as default mode state, or the resting state network (RSN) used to explore functionally segregation of brain regions or network and has become a preferred method for determining neuronal activity by assessing functional connectivity (Fc) in the brain.^5^ The fMRI BOLD technique is extremely useful in acute painful conditions and experimental pain where there are brief episodes of pain followed by brief episodes of pain free periods, resulting in rapidly changing hemodynamic response.^28^ However, monitoring of responses to changes in chronic painful TMD is not well served by this technique.

For chronic painful TMD conditions, an alternative fMRI technique utilizing arterial spin labeling. (ASL) is more appropriate. ASL uses water in arterial blood as a freely diffusible tracer to measure perfusion non-invasively.^29,30^ In contrast to BOLD signal which represent changes in blood flow, vascular volume and oxygen metabolism, this method enables the determination of regional cerebral blood flow (rCBF) as a surrogate marker of neural activity. Generally, these techniques asses both basal neuronal activity and evoked stimuli/task paradigms. The basal neuronal activity refers to metabolic activity of brain tissue occur when a person is awake and not focused on any particular task or experiment. While evoked parameters take measurements of brain activity patterns during administration of stimuli or performance of a particular task.^5,29–31^

In additional to fMRI, structural brain imaging is used to investigate the alterations in anatomic structures in individuals with painful TMD.^32,33^ Structural neuroimaging is based on high resolution T-1 weighted images which are specifically tailored to delineating the boundary of gray and white matter. It can also be used to assess global measures such as whole brain volume, gray and white matter volume as well as regional features such as cortical thickness and voxel-based parameters such as gray or white matter values, interpreted as regional gray/white matter density to volume.^5,34,35^ Additionally, non- invasive measurements of the molecular constituents of in vivo tissue can also be performed. Diffusion tensor imaging (DTI) is another method that can be used in study of chronic pain. DTI measures the white matter by measuring the diffusion of water in the brain such as fractional anisotropic (FA) and mean diffusivity (MD) interpreted as a marker of white matter integrity. DTI can also be used to perform tactography or three-dimensional modeling of neural tracts which can subsequently be used to determine the connections of remote brain regions, providing a structural connectivity marker.^36,37^

While fMRI and sMRI are based on radio frequencies of protons within water molecules, magnetic resonance spectroscopy (H-MRS) used in majority of human imaging studies detects radio frequencies of protons binds to carbon atoms that is organic compounds. This approach enables the quantification of several metabolites/compounds which are assumed to reflect the specific features of neuronal or astrocyte integrity and metabolism.^38^ Typically metabolites such as glutamate (Glu), glutamine (Gln), N-Acetyl Aspartate (NAA), choline (Cho), total creatine (tCr), myoinositol (MI) and lactate are measured.^13^

Over the past few years, much progress has been made in all three areas functional, structural and neurochemical. Since, ASL allows for quantification of regional blood flow and detects regional neural activity and connectivity, future studies may use combination of ASL and BOLD imaging. The combination of these methods enables to better understand how brain activity and vascular responses interact which is of particular importance as neural activity cannot be measured directly by using MRI techniques. Similarly, T1 T2 mapping and magnetization transfer ratio MTR provide more in-depth interpretation of voxel-based parameters and helpful in revealing additional facets of altered microstructure in chronic pain conditions.^5^ Moreover, GABA quantification has been a step forward for H-MRS, enabling for an accurate measurement of the most significant inhibitory neurotransmitter providing indirect evidence of neuronal excitability.^39^

## Pain network

There is no pain center in the brain but instead a complex network of brain regions called as pain matrix are assessed using functional imaging.^40^ Generally, the primary and secondary somatosensory cortex (S1 and S2), insular cortex (IC), anterior cingulate cortex (ACC), the prefrontal cortices (pFC) and the thalamus are the principal part of this pain network. The location of thalamic nuclei is used to infer the nomenclature of the pain system. The lateral system of thalamus project to posterior insula and primary and secondary somatosensory cortex (S1 and S2) which are thought to reflect sensory-discriminative component of pain. While the medial thalamus system which sends signals to limbic structures such as anterior cingulate cortex (ACC), anterior insular cortex and frontal structures represent the affective and motivational component.^41^ These areas are thought of as a multi-sensory integrations site and they have been linked to various aspects of pain related experiences, including anticipatory pain, emotional pain processing and anti-nociception. Therefore, the somatosensory, limbic and associative brain regions are thought to be involved in the pain system.^42–44^ Additionally, the pain system related to TMD also involve the trigeminal nerve root, trigeminal ganglion (TG), spinal tract subnucleus caudalis (spVc), thalamus and somatosensory cortex.^45^ Further, motor and premotor areas and dorsolateral prefrontal cortex (dLPFC) have also been shown to be activated by pain stimuli but these areas are less consistent and it is unclear whether they represent epiphenomenon such as pain-evoked movements, or movement suppression or top down modulation (periaqueductal gray-raphe [PAG] magnus pathway) respectively or directly related to pain perception.^5,20^

**Fig. 2:**
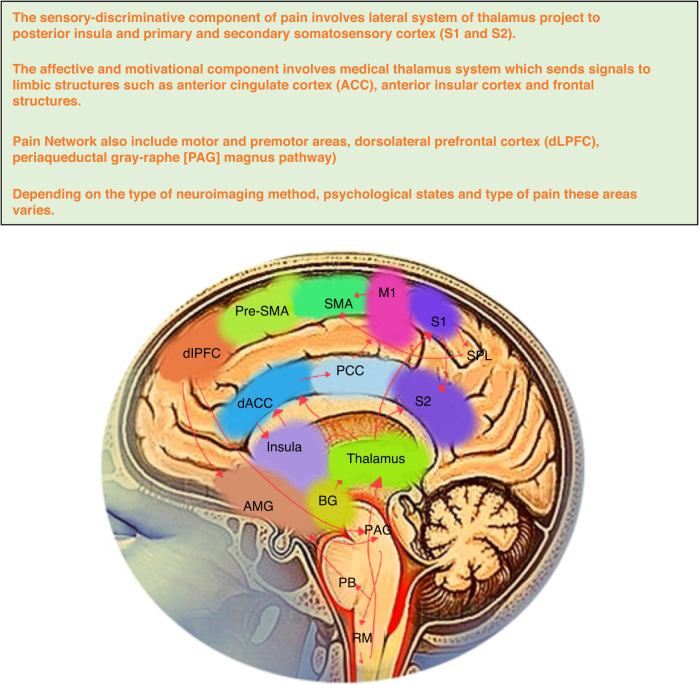
Brain regions and pain network involved in chronic temporomandibular disorders. *AMG* amygdala, *BG* basal ganglia, *dACC* dorsal anterior cingulate cortex, *dLPFC* dorsolateral prefrontal cortex, *M1* primary motor area, *PB* para brachial, *PCC* posterior cingulated cortex, *PAG* periaqueductal gray, *pre-SMA* part of supplemental motor area, *RM* raphe magnus, *S1* and *S2* primary and secondary cortex, *SMA* supplemental motor area, *SPL* superior parietal lobe area

In the context of chronic painful TMD other brain areas such as nucleus accumbens, the hippocampus, the frontopolar regions, amygdala, inferior parietal lobe, superior temporal gyrus, and parietal association cortices have all been found to correlate with particular aspects of pain perception.^21,42,45^ It has been proposed that this network is specific to pain perception decoding discrete elements of pain such as pain intensity and unpleasantness. Additionally, pain matrix activation has also been found in response to many salient and behavioral stimuli. Based on these studies have suggested that different chronic pain disorders seem to be characterized by unique functional and structural brain signatures and the presence of an individualized pain mechanism rather than a rigid pain system. (Fig. 2).

## Neuroimaging studies in painful TMD

This section will provide an overview of MRI neuroimaging studies that reveal structural and functional changes in the brain associated with chronic painful TMD. (Table 2).

**Table 2:** Summary of major neuroimaging MRI studies on TMD

Studies	Analysis method and modality	Major findings
Wilcox et al. ^46,48^	3D T1 and DTI, VBM FA and MD	Regional GMV decrease in medullary dorsal horn with MD increase in patients with TMD. MD changes in the region of PAG and nucleus raphe magnus. Decrease FA in root entry zone of trigeminal nerve, SpVc and ventral trigeminal thalamic tract
Moayedi et al. ^49,50^	3D T1 and DTI, Cortical thickness, VBM FA and MD	In TMD patients increased cortical thickness noted in S1 and PFC. GMV in sensory thalamus positively correlated to TMD duration. TMD had GMV loss, but TMD duration was not correlated to GMV. Positive correlations between orbitofrontal cortex and Neuroticism. Decreased FA in right and left trigeminal tracts. Widespread microstructure alterations of white matter tracts related to sensory, motor and cognitive pain function. FA correlated with TMD characteristics.
Gustin et al. ^51,52^	3D T1, ts-fMRI, DTI and ASL and Whole brain VBM, Brain activation, FA and rCBF	VBM revealed no changes in GMV in TMD. No functional reorganization in the S1 of TMD patients and in rCBF of TMD patients
Younger et al. ^32^	3D T1and whole brain VBM	No overall difference in GMV between TMD and HC. Increased GMV in limbic regions such as posterior putamen, globus pallidus, and anterior IC. Self-reported pain severity was associated with increased GMV in pgACC and PCC
Zhang et al. ^53^	rs-fMRI- FC voxel wise	Decreased regional homogeneity in right anterior IC. Decreased positive FC between right anterior IC and MCC
Ichesco et al.^54^	rs-fMRI and ts-fMRI and FC voxel wise	Increased Fc between left anterior IC and pgACC during both resting and applied pressure state. Negative correlation between functional connectivity and clinical pain intensity
Weissman-Fogel et al. ^56^	ts-fMRI and brain activation and ROI-wise FC	Increased task-evoked responses in brain areas implicated in attention (lateral prefrontal, inferior parietal), emotional processes (amygdala and pgACC), motor planning and performance and activations of the DMN
Kucyi et al. ^57^	rs-fMRI and FC voxel wise	Increased mPFC FC with other DMN networks, including PCC/Pcu, retrosplenial and areas within visual cortex. Pain rumination scores are positively correlated to mPFC FC with the PCC/Pcu, retrosplenial, medal thalamus and PAG
Yin et al. ^59^	fMRI Structural abnormalities and static and dynamic Fc	Decreased cortical thickness in the right sensorimotor cortex, Decreased volume in the left putamen and associated reduced dynamic FC with the anterior midcingulate cortex and alterations in emotion processing and regulation regions including decreased volume/surface area in the left posterior superior temporal gyrus and associated increased dynamic FC
Youssef et al. ^61^	ASL, CBF and brain stem blood flow	TMD patients had increased CBF in regions associated with higher order cognitive and emotional functions
He et al. ^68^	rs-fMRI and FC voxel wise	Decreased fractional amplitude of low frequency fluctuation in left precentral gyrus, SMA and striatum
Salomons et al. ^69^	3D T1 and DTI and VBM, FA and MD	Magnitude of self-related helplessness correlated with cortical thickness in SMA and MCC, regions implicated in cognitive aspects of motor behavior
Gerstner et al. ^70,74^	MRS Metabolite level	Left-insular Gln levels were related to reported pain, left insular NAA levels positively correlated with pain symptom duration. Left insular NAA and Cho levels higher at baseline
Harfeldt et al. ^71^	MRS Metabolite level	Only tCr levels was higher in TMD than controls
Harris et al. ^75^	MRS Metabolite level	Increased level of glutamine in the IC

Data from the published studies^13,23,32,46–75^

### Structural neuroimaging methods in TMD

Structural neuroimaging methods have investigated peripheral and central changes in chronic painful TMD. In a study a decreased trigeminal nerve fiber density, axonal diameter and myelination as well as micro-structural alterations observed in chronic painful TMD.^46^ The investigators, also observed a decreased gray matter volume (GMV) and an increase in rCBF in the ipsilateral spinal sub nucleus caudalis (SpVc) which processes nociceptive input of the TMD patients. They hypothesized that reduction in GMV reflect neuronal loss, while the elevated blood flow in SpVc could be a compensatory response of increased neural activity or hyper excitability due to nociceptive pathways which is critical for the altered perception and maintenance of pain in TMD.^47^ Some studies used DTI which provide measure of water diffusion such as FA and MD to investigate structural alterations in brain stem focusing on the SpVc and TMD pain-processing pathways. Wilcox et al. demonstrated a significant increase in MD in the ipsilateral trigeminal nucleus, and trigeminal tract within the pons and PAG.^48^ Another DTI study, found lower FA in the brain stem white matter along the ascending nociceptive pathways coursing through the thalamus and tracts projecting to sensorimotor cortex, confirming abnormal peripheral input from trigeminal nerve.^49^

In addition to peripheral and brain stem changes, principal brain regions thalamus and S1 also play crucial role in the thalamocortical pathway related to TMD pain. In a study the duration of painful TMD was found to be positively correlated with alterations in thalamus GMV.^50^ It is hypothesized that this occurs as a result of persistent trigeminal nociceptive input, which promotesTMD hyperalgesia by facilitating trigeminal sensory information from thalamus to S1. Furthermore, structural MRI studies of S1 changes in painful TMD have demonstrated less consistent findings with different studies reporting decreased GMV, increased cortical thickness or no change. This disparity may be due to differences in pain duration as well as the impact of medication.^51^

Gustin et al., used multiple MRI modalities (ts-fMRI, DTI and ALS) to determine whether S1 reorganization occur in painful TMD or not. No functional reorganization in the S1 of TMD patients as well as no significant differences in FA and cBF within the S1 are noticed compared to healthy controls. However, the authors did not investigate the changes in other critical areas as the SpVc and thalamus. Therefore, further studies focusing simultaneously on the SpVc, thalamus and S1 are required to resolve the issue of inconsistencies of S1 changes in TMD patients.^52^

Despite some discrepancies in the literature, the structural and functional findings support the involvement of abnormal of medial pain system in nociceptive processing, demonstrating emotional sensory signals associated with TMD pain. Younger et al., evaluated female patients with myofascial pain in their study and observed an increased GMV in the anterior insula and a negative correlation between self-reported pain and GMV in pregenual ACC and posterior cingulate cortex (PCC).^32^ Similarly studies investigated painful TMD patients’ functional connectivity (Fc) between insula and cingulate cortex and MCC. Zhang et al. revealed a decreased homogeneity in insula in female patients with synovitis compared to healthy controls.^53^ While Ischesco revealed enhanced Fc between insula and pregenual ACC in resting state.^54^ In both studies Fc is negatively correlated with subjective pain intensity. This implies that patient with higher connectivity reported lower pain suggesting compensatory brain changes to control pain.

### Functional neuroimaging methods in TMD

Functional neuroimaging methods have investigated central changes in chronic painful TMD. Default mode network (DMN) is a group of functionally interconnected brain regions that get activated during mind wandering and not involved in any specific task and becomes correspondingly deactivated during goal-oriented tasks has been studied in previous studies.^55^ Studies suggested that dysfunction of DMN may be related to cognitive and behavioral deficits observed in patients with chronic painful TMD^55^. In TMD patients, Weissman-fogel et al. observed task-evoked activity in the PCC and medial prefrontal cortex as well as functional disconnections throughout the DMN. The increased activation of PCC may reflect increased spontaneous pain in TMD patients as cognitive activities with emotional interference elicited more emotional effects on patients than control.^56^

Moreover, Kuyci et al. linked dysfunctional DMN with pain rumination in TMD and observed improved Fc between mPFC and other DMN networks (retrosplenial cortex, PCC/precuneus and portion of visual cortex). This suggested that individuals with high pain rumination had particularly enhanced Fc.^57^ In addition, the positive correlation observed between pain rumination and Fc of medial prefrontal cortex (mPFC), thalamus and PAG which regulate endogenous pain modulation.^58^ Since, healthy controls had no such correlations it has been suggested that the extent to which chronic painful TMD alters the normal function of these circuits may be determined by how much patients ruminate. Thereby highlighting the crucial role of pain related cognition in TMD-related brain changes. As most study detected static Fc, recently another study examined static and dynamic Fc as well as brain regions with structural abnormalities in patients with chronic painful TMD. The research group identified a decreased cortical thickness in right sensorimotor cortex and decreased volume in left putamen and associated reduced dynamic Fc with anterior midcingulate cortex and alterations in emotion processing and regulation regions for instance decreased volume/surface area in the left posterior superior temporal gyrus and increased dynamic Fc with precuneus in TMD patients.^59^ Since the structural and functional abnormalities in brain regions implicated in sensorimotor and emotional functions the above discussed study provided evidence for the biopsychosocial model of TMD and facilitated our understanding of the mechanism underlying TMD. In other study researchers also concluded that TMD patients who have negative emotions may have dysfunction within the reward system as well as painful TMD patients may have dysregulated spontaneous activity and Fc in the DMN, sensorimotor network and pain related regions, fronto-striatal-limbic circuits respectively.^60^

Furthermore, cingulo-frontal regions, amygdala, and hypothalamus have all been shown to be involved in chronic painful TMD. Patients with TMD often show cognitive abnormality. Increased rCBF observed in TMD patients in the ACC, dLPFC, and precuneus (Pcu) areas of brain associated with cognitive and affective activities.^61^ Chronic pain has also been shown to induce attentional biases and attentional manipulation can modulate the perception of pain.^62^ In a study TMD patients showed slower task responses, with reduced Fc between two pairs of brain regions such as aMCC- dlPFC and pgACC-Amygdala.^32^ Reduced connections between these two pairs of structures in TMD patients may indicate a chronic pain influence on attentional (aMCC-dlPFC) and emotional network (pgACC-Amygdala) resulting in attenuated and un synchronized recruitment of attention processing areas and consequently slower response.^63,64^ In another study, cortical thickness was measured in TMD patients. Researchers reported that TMD patients had thicker cortex in the frontal pole and ventrolateral prefrontal cortex (vlPFC) compared with control and cortical thickness in ventromedial prefrontal cortex (vmPFC) which is part of orbit-frontal-cortex is positively correlated with neuroticism scores in TMD patients.^50^ This finding in conjunction with neuroticism suggests that higher level of distress or anxiety in TMD patients are linked to changes in the vmPFC, which may result from or contribute to a decreased brain capacity for pain control.^23,65^ Since there is positive association between ADHD and occurrence of painful TMD symptoms a recent study has investigated brain dynamics and observed alteration in Fc within DMN and sensorimotor regions.^66,67^

There are studies which have revealed changes in motor system including primary motor cortex (M1), supplementary motor areas (SMA) and striatum in patients with painful TMD.^68,69^ It is observed that persistent pain can inhibit protective movement and impair motor performance due to maladaptive neuroplasticity in motor cortex. Wessman-Fogel et al., observed enhanced activity in M1 and SMA areas in TMD patients during cognitive interference Stroop task which may be due to compensatory mechanism to recruit more motor areas to meet demand of motor performance and planning.^56^ Additionally, chronic pain can result in learned helplessness, a maladaptive response characterized by decreased motor escape behavior and deficit in motivation and learning. Salomons et al. reported no significant group difference between cortical thickness of SMA nor correlation with pain characteristics such as self-reported helplessness in TMD patients.^69^ In addition to M1 and SMA, the striatum has also been implicated in the motor response to pain in TMD. The striatum receives input from cortical regions and thalamic nuclei and sends output to other basal ganglia structures serving as a critical site where cognitive, motor and limbic signals from other brain regions overlap or integrated. Studies on TMD patients observed an increased GMV in putamen, globus pallidus and striatum relative to control.^70^ This suggest behavioral response preparation to nociceptive stimuli.

### Magnetic resonance spectroscopy in TMD

Several studies have shown that painful TMD is associated with changes in the brain metabolism. It is observed that NAA is a marker of neuronal health and density, Cho is related to increased cell numbers, membrane synthesis and membrane break down. tCr is considered as an important metabolite of cell energy and metabolism. Glutamine (Gln) is metabolite of glutamate (Glu), together they participate in complex metabolic process and intercellular communication involving neurons and astrocytes.^71,72^ MI is primarily present in glial cells and plays an important role in osmoregulation.It is observed that few of these neurochemicals can be used to assess the underlying mechanism of TMD pain.^72–74^ In a study, Gerstner et al., found a negative correlation between Gln levels in the left insula and pain in TMD patients while NAA and Cho levels in the left posterior insula were increased compared to healthy controls. In this study NAA levels were positively correlated with the duration of pain. As NAA is considered as measure of neuronal health and synaptic integrity, it has been postulated that a time dependent neuronal growth occurs in response to TMD pain.^75^ Similarly, a state of neuroinflammation and cellular hyperactivity that has been postulated to be associated with chronic pain may also be indicated by the elevated tCr levels in the posterior insula in TMD patients, since tCr can be considered of as a measure of cell energetics.^71^ Harris et al. demonstrated in the context of chronic pain that patients with fibromyalgia had increased glutamine level in the IC and that variations in posterior insular glutamate corresponds with variations in both experimental pain thresholds and clinical pain.^75^

These findings provide new evidence about the involvement of the neurochemicals as a measure in the neurobiology of underlying TMD. It is also a further step towards understanding and accepting that this measure can be used to assess the orofacial pain mechanism.^76,77^ However, need to be further tested for replicability and validity. Since TMD encompasses painful conditions of muscle, joint and associated structures, the large variability in diseases characteristics of patient groups precludes drawing firm conclusion about brain changes in TMD with a specific type and pain origin. Also, the paucity of large sample sizes and variety of methodological approaches limit the process of synthesizing findings to draw broad generalizations.

## Artificial Intelligence in the diagnosis of TMD

**Fig. 3:**
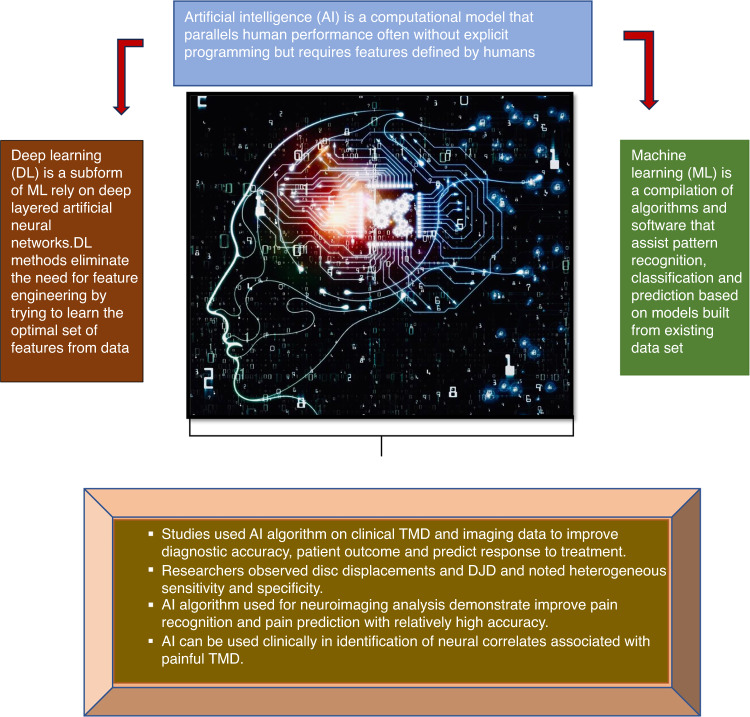
Overview of AI on assessment of chronic painful temporomandibular disorders

Due to multifactorial etiology, comorbid conditions and various symptoms presentation diagnosis of the TMD is challenging. Therefore, for diagnosis of TMD a detailed history, and clinical examination and imaging is required. AI has been recently deployed to detect and quantify TMD.^78^ In order to emulate the human decision making and reasoning process AI aims to encode intelligence into machines by learning from experiences and adapting to changes in the environment. In past few years various studies have used AI to enhance clinical judgment and facilitate diagnosis.^26,78–82^ The foundation of AI is machine learning which is concerned with algorithms that are capable of learning complex tasks and developing predicting models through sample data. Similarly, deep learning is class of artificial neural networks (ANN) that eliminate the need of feature engineering by trying to learn the optimal set of features from data.^25,83–85^ (Fig. 3).

In last decade, there is growing interest in developing data-driven, actionable strategies that can personalized assessments, diagnosis, prognosis and treatments of individuals with painful TMD.

So far, the Diagnostic Criteria for Temporomandibular Disorders (DC-TMD) is the widely accepted criteria used by the clinicians and researchers.^9^ It comprises of two axes, Axis-I and Axis-II, which include diagnostic standards for differentiating painful TMD and intra-articular disorders and assessing jaw function, behavioral and psychosocial factors. However, for a few intra-articular disorders DC-TMD has limitations in terms of diagnostic accuracy. For instance, disc displacement with and without reduction and locking demonstrate low sensitivity (0.34-0.54). Similarly, degenerative joint diseases demonstrate low sensitivity and specificity of 0.55 and 0.61 respectively. Further the inter-examiner reliability is low for disc displacement and degenerative joint diseases (DJD.^9^ Often use of screening tools to determine patient’s symptoms are time consuming and place a burden on clinicians to predict the outcome. Hence, investigators have used AI to analyze TMD data to improve diagnostic accuracy, patient monitoring and develop new protocols.^25,86^ (Table 3).

**Table 3:** A summary of artificial intelligence studies on painful TMD

Summary of major artificial intelligence studies on TMD		
Studies	Study objective	Inference
Bas et al.^86^	Use of artificial neural network (ANN) for diagnosis of temporomandibular joint internal derangement and normal joints	Heterogenous sensitivity and specificity noted for the diagnosis of disc displacements using ANN. The application of ANN for diagnosis of subtypes of TMJ internal derangement may be a useful supportive diagnostic method
Radke et al.^87^	ANN for detection of normal TMJ and non-reducing displaced disks	The ANN detected the presence and type of non-reducing disk displacement from frontal plane recordings of gum chewing in a group of real patients seeking treatment
De Dumast et al.^88^	Deep neural network to assess shape changes in TMJ osteoarthritis (TMJOA) and web-based system for neural network classification of TMJ OA	Study demonstrates a comprehensive phenotypic characterization of TMJ disease at clinical, imaging and biological levels using web-based system that provides advances shape statistical analysis and a neural network-based classification of TMJ OA
Bianchi et al.^89,93,95^	Diagnosis of TMJOA using quantitative bone imaging biomarkers and machine learning	Total 13 imaging biomarkers presented an acceptable diagnostic performance fir diagnosis of TMJ OA using CBCT AND accuracy of 0.823 observed for diagnosis of TMJ OA status
Kim et al.^91^	Automated detection of mandibular condyle using convolutional neural networks (CNN) and faster region-based CNN (R-CNNs)	The sensitivity specificity and accuracy of the TMJOA classification algorithm using CNN are 0.54, .94 and 0.84 respectively and classifying panoramic images using CNN is possible
Shoukri et al.^92^	Test correlation of biomarkers of condylar morphology and find deep neural networks to assess bony changes in TMJ OA	Study demonstrates a significant correlation among variations in protein expression levels, clinical symptoms and condylar surface morphology. The results suggest that 3-dimensional variability in TMJ OA condylar morphology can be comprehensively phenotyped by the neural networks
Reda et al.^27^	Present the AI based system for supporting non-expert dentist in early TMD recognition	Study provides a preliminary proof of concept of the feasibility of implementing an AI based system in early identification of TMDs.

Data from assorted studies^25,27,85–94^

There are studies that used machine learning to address painful TMD disorders.^25,86–88^ Researchers observed DJD, osteoporosis, disc displacements with and without reduction and found heterogenous sensitivities and specificities.^87–90^ Some studies also used deep learning algorithms and image data to diagnose DJD using CBCT images.^78,79^ Lee et al. developed an automated diagnostic tool for detecting DJD.^78^ Kim et al. used panoramic imaging data which had poor sensitivity and reliability in detecting DJD. Although panoramic is not a standard imaging, AI model demonstrate sensitivity and specificity of 0.54 and 0.94 for diagnosing bony abnormality.^91^ A few studies used machine learning methods to examine correlations between the biomarkers and condylar changes to increase diagnostic sensitivity.^89–93^ Additionally, for analyzing condylar shape alterations in DJD, CBCT image data and AI models were used. The AI model demonstrate accuracy of 80-90% indicating high reliability which is similar to the previous studies.^94–97^

For diagnosis of disc disorders MRI imaging are most frequently used. Bas et al. used clinical symptoms and diagnosis to predict the subtype of disc disorders using artificial neural network and observed varied diagnostic accuracy.^86^ The above studies used AI to support clinicians in diagnosing TMDs using various type of data for instance diagnostic images, health records and biomarkers which may contribute to increase diagnostic accuracy. However, in TMD accuracy of developed model greatly varies one due to complex spectrum disease and two depending on the data used, size and algorithms used for developing the model.

Several other evidence suggested that AI could improve pain recognition and facilitate the use of clinical documents with pain assessment information to identify pain automatically. Computational tools may detect patients pain status from clinical documentations automatically but not real time pain.^83^ Also these tools do not have capability to detect specific quality of pain the area which needs further research. AI could also facilitate pain prediction and improve clinical decision support. It has been shown that ML approach can be used to identify key questions in pain questionnaire to predict pain persistence with relative high accuracy.^98^ Further research is essential to investigate this model and test this approach in more patients and different types of pain. Additionally, AI-based apps were observed to have positive effects on pain management including reducing pain level, reducing the use of interventions and assisting therapy.^83,98–100^ However, the generalizability of these results is subject to certain limitations such as studies only assessed the general pain level instead of the pain at each specific site. Furthermore, studies were limited to immediate post intervention effects of AI based apps, it was impossible to know sustained effects of those interventions.^99,101^ Therefore future research is needed to determine if improvements in pain level could lead to changes in other functions or other long term physiological changes. Further work is required to establish the viability of these novel systems and test different combination of technologies. Moreover, comparison with an appropriate reference standard should be considered in future research.

### Artificial intelligence and neuroimaging methods for TMD

Despite these encouraging findings it is important to point out on our understanding of chronic painful TMD and the underlying neurobiological mechanisms. Given this, a combination of neuroimaging and AI may benefit investigators in understanding the mechanism of chronic painful TMD. A growing number of neuroimaging studies have shifted from establishing general neural activation patterns or connections related to specific tasks or behaviors to uncover individual level variations that can be used to make predictions about an individual’s behavior.^101,102^ ML has been a critical component in this advancement. ML can identify patterns and relationships of signals in neuroimaging data.^103^ It can also help in quantification of anatomy as well as in detection and analysis of findings associated with painful TMD.

**Fig. 4:**
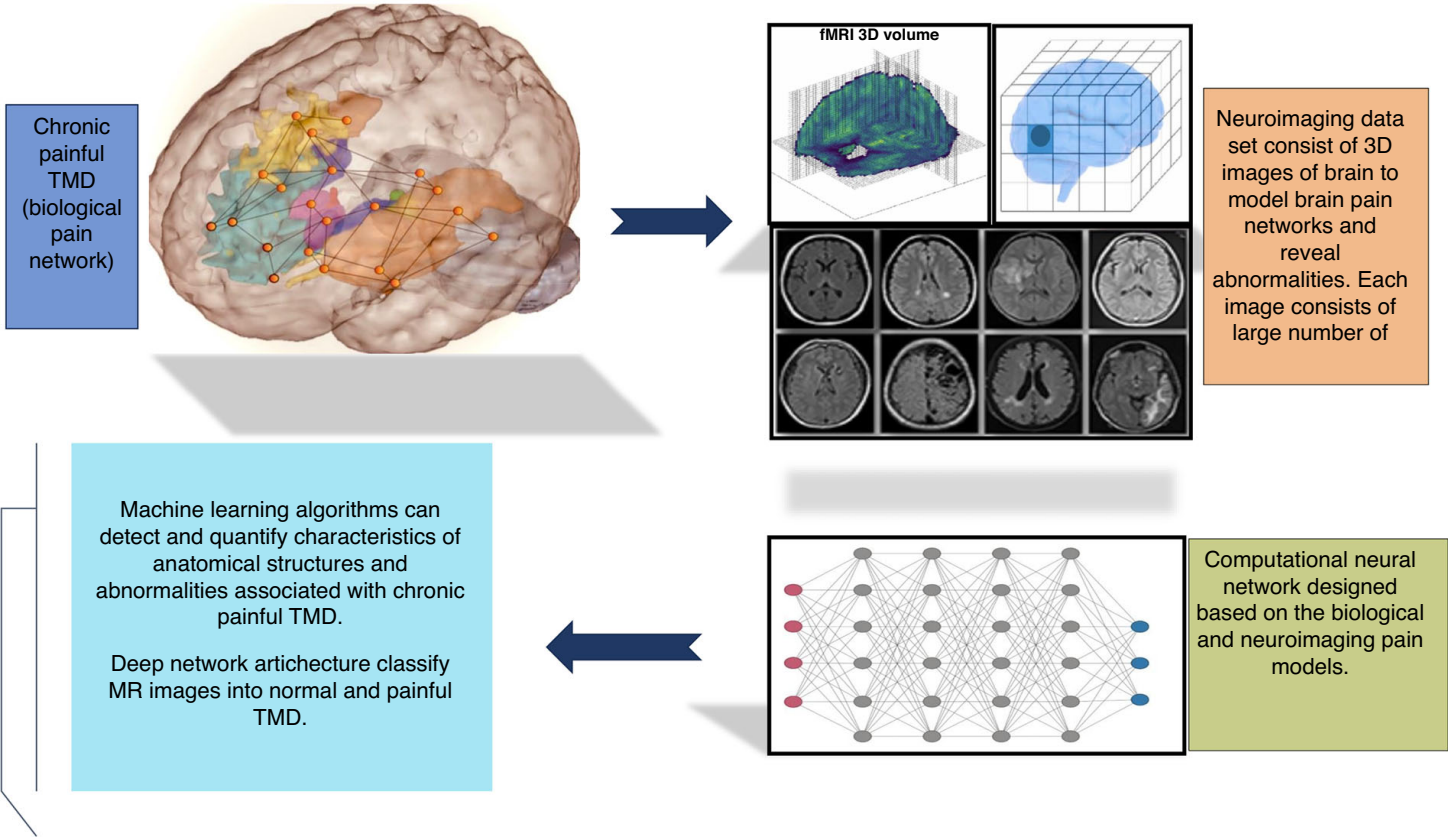
Relationship between neuroimaging and machine learning algorithms

Generally, supervised and unsupervised learning are two types of ML methodologies. In supervised ML some neuroimaging data exists which is used to train the algorithms.^104^ For example, collection of neuroimaging data that a neuro-radiologist has classified into painful TMD and non-painful TMD groups. In contrast for unsupervised learning, no criterion standard images or classifications are used, and the computer must itself determine the classes. Unsupervised ML identify patterns in a large set of observations establishing normal variability or spotting groups of related observations. For assessment of chronic painful TMD, both supervised and unsupervised ML method are appropriate. Deep learning approaches are required to understand the neural pain networks in chronic painful TMD. The DL model is a specified learning method typically inspired from how the human brain or biological pain network is structured. It consists of multiple layers of artificial neural networks which corresponds to a pain network observed in neuroimaging data.^103,104^ (Fig. 4).

To date multitude of ML approaches have been applied to identify and characterize the brain lesion. The ML algorithms detect the neuroimaging data such as alterations in cortical thickness based on different voxel intensities or BOLD signal detected from each voxel image of fMRI to identify brain areas whose signals is associated with chronic painful TMD.^103,105^ In structural imaging data, ML facilitates quantifying size of brain structures and their deviation associated with underlying pain disorder and act as potential markers for clinical outcome. As the number of structural images of patients with painful TMD and controls increases, ML will generate more robust models for segmentation, classifications and prediction tasks.^106^ While in functional imaging, Ml algorithms are used in univariate (imaging each brain region) and multivariate fashion (imaging entire brain region to analyze relationships different brain regions) which aim to identify brain regions whose functional signal is associated with the condition. Additionally deep learning-based approaches have shown promising results in both speed and accuracy in segmentation of neuroanatomy compared to standard tools as well as in establishing markers of diseases with prognostic values.^103–107^

**Fig. 5:**
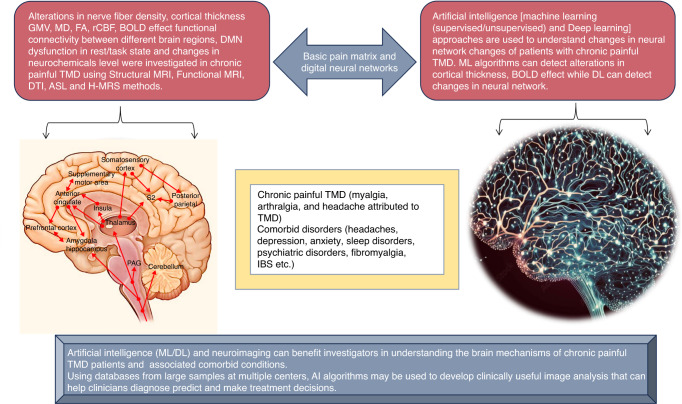
A summary on role of neuroimaging methods and AI in chronic painful TMD

Finally, brain networks architecture is critical for cognitive capabilities, and it can be affected by chronic pain.^103^ Hence, quantifying and understanding associated changes in network architecture or model reorganization mechanism associated with pain progression and recovery are clinically relevant.^108^ Moreover, chronic painful TMD occur generally in association with other psychological and sleep disorders. ML algorithms can also be clinically useful in exploring the neural mechanism of underlying psychiatric, psychological and sleep disorders.^109^ In a selective review the application of ML in psychoradiology was discussed. According to the review the ML not only helps in identifying the disorders specific brain functional dynamics but also offered biomarkers for precise diagnosis and personalized treatment.^110^ As a result it is crucial to use ML to understand the deviation in connectivity from normal networks and exploring specific reorganization patterns of network architecture underlying chronic painful TMD and associated psychiatric and sleep disorders. Further, ML also helps in identification of association between chronic pain and network alterations as well as in identification of early biomarkers which can predict transition from acute to chronic pain. More experimental and clinical studies can provide valuable opportunity to stud the brain structural and functional abnormalities, which may help us obtain more information about the genesis of chronic pain. (Fig. 5).

### Limitations

Although neuroimaging methods has had a significant impact on our understanding chronic painful TMD, most studies in the literature focus on establishing group differences or qualitative and quantitative measures of pain such as pain intensity, duration etc. so drawing conclusion about subject specific information is difficult. Additionally, TMD encompasses different pain conditions as Myalgia, arthralgia, and headaches the large variability in TMD characteristics of patients’ groups precludes drawing a firm conclusion about brain changes in TMD with a specific type and pain origin. To address this issues studies aimed at identifying patient’s subgroups in large samples of TMD group are required. Similarly challenges related to data screening, collection and data verification have consistently been reported. Given that TMD is heterogenous disorder associated with other comorbid conditions understanding the personal difference is critical for choosing a proper clinical management approach. Therefore, a combination of neuroimaging techniques and machine algorithms such as SVM, DT And ANN may serve to benefit future studies. The ML algorithms can be used to not only build predictive systems for diagnosis and prediction but also to advance and deepen our understanding of underlying biological mechanism of painful TMD. However, a recent review identified limitations of existing ML approaches in health care such as inability to consistently perform across the size and variety of data within health care.^111^

## Conclusion and future perspectives

The application of neuroimaging has significantly improved our understanding of chronic painful TMD and the underlying neurobiological mechanism (Table 4. Clinical Points). This review demonstrates different neuroimaging methods and existing literature on brain changes in patients with TMD. The major observation is that the central nervous system of chronic painful TMD patients demonstrate changes in brain function, structure, and chemistry. MRI have provided a deeper understanding of what happens to the brain structure and function in TMD patients which provide both physiological and pharmacological targets for us to collectively develop. The cellular mechanism under these changes still needs to be explored. Increase multidisciplinary collaboration, combine with more precise tools for understanding responses within the pain matrix, should result in the development of new and more effective chronic pain therapies in the coming decades.

**Table 4:** Clinical points

Clinical points
(1) Chronic pain is a global health problem. Patients with TMD show patterns of altered brain function, structure and chemistry.
(2) Neuroimaging methods have shed light on disentangling the neural mechanism that underlies TMD.
(3) Artificial Intelligence plays an important role in the exploration of neuroimaging data related to TMD.
(4) Future AI may investigate imaging data with additional clinical data from the record to improve accuracy in the diagnosis and prognosis of TMD.
(5) Neuroimaging techniques particularly when combined with artificial intelligence, genetic and molecular approaches may have significant impact on the diagnosis and differentiation of painful TMD as well as the evaluation of the efficacy of therapeutic interventions.
(6) Further research is required to determine whether chronic painful TMD shares a common brain signature or if different types of painful TMD can ultimately be classified by their unique pathophysiology.
(7) In the future, with standardization and validation, neuroimaging and AI could provide objective biomarkers of chronic painful TMD, and guide treatment for personalized pain management.

AI is becoming increasingly important in the use of neuroimaging data for research and clinical applications. This paper also discuss the AI algorithms developed for TMD diagnosis that can be used by the clinician to support decision. More diagnostic images features and various input data may aid in improving TMD diagnostic accuracy. ML current clinical relevance is based on its ability to detect, quantify and compare neuroanatomy and disease related patterns. ML methodology can bridge the gap between representing imaging data and other molecular markers of painful TMD. Most previous studies lack external validation and certainty of evidence was very low. Further studies with larger data set to ensure generalizability of develop models are warranted.
